# International chemistry for a sustainable society

**DOI:** 10.1093/nsr/nwab038

**Published:** 2021-03-09

**Authors:** Zhigang Shuai

Since 1978, thanks to Deng Xiaoping's open policy, China's story has been a big success: not only in becoming a world economy power, but also for great advancements in science and technology. Such great success has led China from isolation to become an active member of the global village. China benefited not only from capital investments and modern management, but also science and technology from the developed world. In only 40 years, China has made a great leap forward to become a world economic locomotive.

Nevertheless, ever since the Industrial Revolution, world developments have led to alarming depletion of resources and irreversible pollution. As far as chemistry is concerned, Chinese chemists are ready to share the international responsibility and to work closely with international colleagues to tackle the global challenges in energy, sustainability, water, materials and health issues. The Chinese Chemical Society (CCS) consecutively led by Prof. Chunli Bai and then by Prof. Jiannian Yao, has initiated several inter-society memoranda of understanding, for example, with the American Chemical Society (ACS), German Chemical Society (GDCh), Royal Society of Chemistry (RSC) of the UK, French Chemical Society (SCF) and more. The CCS is also an active member of the International Union of Pure and Applied Chemistry (IUPAC) and Federation of Asian Chemical Societies. Prof. Qifeng Zhou (former President of Peking University) served as IUPAC President in 2018–2019.

As early as 2003, under encouragement from then CCS President Prof. Chunli Bai and CCS Secretary-General Prof. Jiannian Yao, I started to work as a volunteer for the international affairs of CCS. One of my initiatives was to co-found a consortium ‘CS3’ (Chemical Sciences and Society Summits) with other major chemical societies supported by the funding agencies of the respective countries. The motivation was to unite the forces of international chemists to propose a roadmap of chemistry solutions to meet the global challenges. So far, we have issued eight white papers covering solar energy, sustainable materials, health issue, element strategy, water, etc. The latest one is ‘Science to enable sustainable plastics’. These white papers have provided guidelines for funding agencies around the world, in addition to making public appreciation of chemistry for sustainability.

It should be noted that the pandemic of COVID-19 has largely devastated the world economy and hindered international travel. But the international scientific community had long reached the consensus that only international cooperation can solve the global problems, including the present difficulty. This is the purpose of this special topic of NSR on ‘International Chemistry for a Sustainable Society’, in which the international chemistry leadership shares their opinions as well as practices for international cooperation. We are confident that chemistry can provide solutions to tackle global challenges in energy, resources, pollution, water and many other areas.

***Conflict of interest statement***. None declared.

